# Skeletal Mineralization in Association with Type X Collagen Expression Is an Ancestral Feature for Jawed Vertebrates

**DOI:** 10.1093/molbev/msz145

**Published:** 2019-07-04

**Authors:** Mélanie Debiais-Thibaud, Paul Simion, Stéphanie Ventéo, David Muñoz, Sylvain Marcellini, Sylvie Mazan, Tatjana Haitina

**Affiliations:** 1 ISEM, Université de Montpellier, CNRS, IRD, EPHE, Montpellier, France; 2 The Neuroscience Institute of Montpellier, Inserm UMR1051, University of Montpellier, Saint Eloi Hospital, Montpellier, France; 3 Department of Cell Biology, Faculty of Biological Sciences, Universidad de Concepción, Concepción, Chile; 4 Sorbonne Universités, UPMC, CNRS UMR7232 Biologie Intégrative des Organismes Marins, Observatoire Océanologique, Banyuls-sur-Mer, France; 5 Department of Organismal Biology, Uppsala University, Uppsala, Sweden

**Keywords:** type X collagen, chondrichthyan, mineralization, teeth, scales

## Abstract

In order to characterize the molecular bases of mineralizing cell evolution, we targeted type X collagen, a nonfibrillar network forming collagen encoded by the *Col10a1* gene. It is involved in the process of endochondral ossification in ray-finned fishes and tetrapods (*Osteichthyes*), but until now unknown in cartilaginous fishes (*Chondrichthyes*). We show that holocephalans and elasmobranchs have respectively five and six tandemly duplicated *Col10a1* gene copies that display conserved genomic synteny with osteichthyan *Col10a1* genes. All *Col10a1* genes in the catshark *Scyliorhinus canicula* are expressed in ameloblasts and/or odontoblasts of teeth and scales, during the stages of extracellular matrix protein secretion and mineralization. Only one duplicate is expressed in the endoskeletal (vertebral) mineralizing tissues. We also show that the expression of type X collagen is present in teeth of two osteichthyans, the zebrafish *Danio rerio* and the western clawed frog *Xenopus tropicalis*, indicating an ancestral jawed vertebrate involvement of type X collagen in odontode formation. Our findings push the origin of *Col10a1* gene prior to the divergence of osteichthyans and chondrichthyans, and demonstrate its ancestral association with mineralization of both the odontode skeleton and the endoskeleton.

## Introduction

Several factors make type X collagen an excellent candidate to study the evolution of the genetic mechanisms involved in skeletogenesis. First, although type X collagen was first identified more than 30 years ago in chicken long bones (Schmid and Linsenmayer 1985), it has to date only been described in osteichthyans. An immunohistochemical analysis using rabbit anti-Col X antibodies labeled the shark skeleton (Seidel et al. 2017), but the elephant shark and whale shark genome sequencing projects have failed to identify type X collagen genes (Venkatesh et al. 2014; Read et al. 2017). Second, type X collagen is a crucial factor of skeletal mineralization in amniotes, expressed in hypertrophic chondrocytes during endochondral ossification (Schmid and Linsenmayer 1985; Jacenko et al. 1993; Mcintosh et al. 1994; Kwan et al. 1997), and contributes to the initiation of calcium precipitation in the cartilaginous extracellular matrix (Kirsch and Wuthier 1994). Third, studies in nonamniote osteichthyans have revealed that *Col10a1* is not limited to cartilage mineralization as it is expressed by osteoblasts in the amphibian *Xenopus tropicalis* (Aldea et al. 2013) and the actinopterygians: the medaka *Oryzias latipes* (Renn and Winkler 2010), the spotted gar *Lepisosteus oculatus*, and the zebrafish *Danio rerio* (Laue et al. 2008; Albertson et al. 2010; Eames et al. 2012). This suggests that in osteichthyans, type X collagen was originally involved in the mineralization of both the cartilage and bone matrix of the endoskeleton. Finally, *Col10a1* originated through the two rounds of genome duplication that occurred early in vertebrate evolution, together with its paralogous *Col8a1* and *Col8a2* genes (Aldea et al. 2013). This suggests that *Col10a1* should have been ancestrally present in chondrichthyans, and raises the question of when it was recruited to the skeletal mineralization processes.

Chondrichthyans or cartilaginous fishes (Elasmobranchii and Holocephalii) have lost perichondral bone and all large dermal bones (Janvier 1996). Chondrichthyan skeletons, however, are characterized by several types of mineralized tissues: globular mineralized cartilage also found in fossil jawless vertebrates (Ørvig 1951; Dean and Summers 2006) and prismatic cartilage which is a chondrichthyan-derived feature (Seidel et al. 2016). A third type of mineralization is only found in elasmobranchs (sharks, rays, and skates), in the fibrous tissue surrounding their notochord, the site of areolar mineralization (Arratia et al. 2001; Eames et al. 2007; Enault et al. 2015; Criswell et al. 2017; Atake et al. 2019). Finally, a fourth mineralized tissue was described in the neural arches of few elasmobranch species: it resembles lamellar bone, and will be here-in named “lamellar mineralization” as its homology with bone in osteichthyans is still debated (Wurmbach 1932; Lallier et al. 1982; Peignoux-Deville et al. 1989; Eames et al. 2007; Enault et al. 2015). Nevertheless, the site of densest mineralization in past and extant vertebrates is found in their odontode skeleton, which includes teeth, scales, and fin spines (Qu et al. 2015). These structures are made of enamel or enameloid outer layer and a dentin core produced as a result of epithelial–mesenchymal interaction, where ameloblasts of epithelial layer secrete enamel matrix and odontoblasts of mesenchymal layer secrete dentin matrix (Hall 2015).

Here, we report the identification of several type X collagen genes in both clades of cartilaginous fishes, elasmobranchs (including the lesser spotted catshark *Scyliorhinus canicula*, whale shark *Rhincodon typus*, thornback ray *Raja clavata*, little skate *Leucoraja erinacea*, thorny skate *Amblyraja radiata*) and holocephalans (represented by the elephant shark *Callorhinchus milii*). We conclude that these type X collagen genes arose from a series of local tandem duplications that occurred prior to the divergence of the holocephalan and elasmobranch lineages, in the time period when the unique hard-tissue repertoire of chondrichthyans evolved. The major expression site of these type X collagen genes in the cartilaginous fishes is in the teeth and scales of the odontode skeleton, whereas only one gene duplicate is also expressed in vertebral mineralizing tissues of the endoskeleton. We further show that a type X collagen expression site is retained in teeth of two osteichthyans, the zebrafish *D. rerio* and the western clawed frog *X. tropicalis*. Our results indicate an ancestral shared involvement of *Col10a1* genes in the formation of a mineralized odontode skeleton, in addition to its role in endoskeletal mineralization, as early as the last common ancestor of all jawed vertebrates.

## Results

### Duplication of *Col10a1* Gene in Holocephalan and Elasmobranchs

We searched the whole elephant shark genome assembly and identified five *Col10a1* genes on scaffold 71, one *Col8a1* gene on scaffold 42, and one *Col8a2* gene on scaffold 121. Using these predicted coding sequences (CDS) to screen transcriptomic data of other chondrichthyan species led to the identification of six similar coding sequences in the catshark *S. canicula* and thornback ray *Ra. clavata*, as well as a single *Col8a1* and *Col8a2* sequence. In the transcriptome of little skate *L. erinacea* we have identified partial gene transcript sequences LSb2-ctg50203 for *Col10a1.1*; LSb2-ctg2668069 for *Col10a1.2*; LSb2-ctg41083 for *Col10a1.4*; LSb2-ctg68055 for *Col10a1.5*; LSb2-ctg688724 for *Col10a1.6*; LSb2-ctg44808 for *Col8a1*; and LSb2-ctg1616013 for *Col8a2*.

**Fig. 1.**
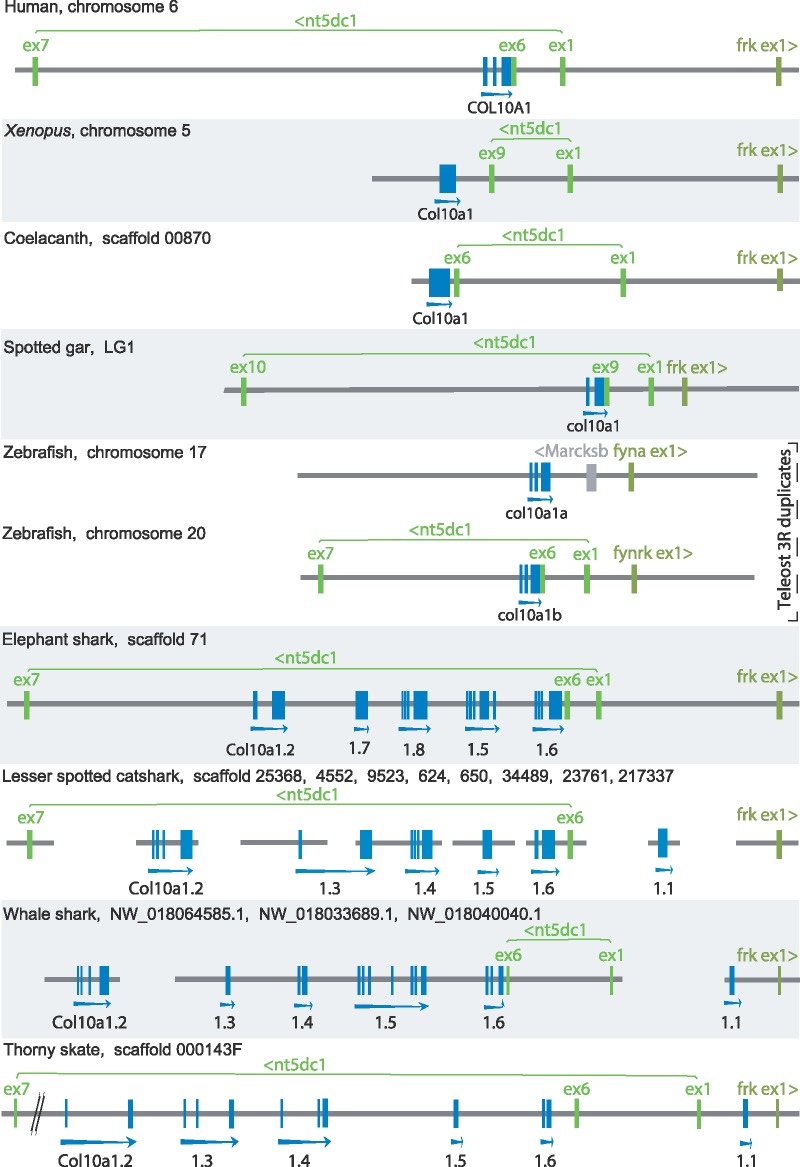
Conserved synteny between *Col10a1* genomic region in osteichthyan species: human, *Xenopus*, coelacanth, spotted gar, zebrafish; and region containing *Col10a1* gene duplicates (numbered .1–.8) in chondrichthyan species: elephant shark, catshark, whale shark, and thorny skate. Genomic data for osteichthyan species were extracted from public databases (Ensembl, NCBI, XenBase) and analyzed to reconstruct the synteny blocks of duplicated *Col10a1* genes in chondrichthyans. Distances are to scale within each species but may differ between species. Abbreviation: ex, exon.

In whale shark genome assembly we identified one of the hits, which was a long predicted transcript (XM_020514194.1) spreading over 100 kb on the genomic locus NW_018064585.1, to which four of the catshark cDNAs could be aligned in tandem (fig. 1 and supplementary table 1, Supplementary Material online). Two additional predicted CDS had a positive reciprocal BLAST best hit with catshark *Col10a1* copies on two other genomic loci (fig. 1). Together, we identified six *Col10a1*, one *Col8a1*, and one *Col8a2* gene sequences in the whale shark genome.

### Conserved Synteny of *Col10a1* Genes in Jawed Vertebrates

The human genomic locus of the *Col10a1* gene (Ensembl ENSG00000123500) is located in the 103 kb long intron 6 of the *5′-nucleotidase domain containing 1* gene (*Nt5dc1*, Ensembl ENSG00000178425). These two genes are located on the opposite strands and a *Fyn related Src family tyrosine kinase* gene (*Frk* ENSG00000111816) has the same orientation as *Col10a1* but located 40 kb away from the 5′ end of the *Nt5dc1* gene (fig. 1). Conserved synteny is described in the *Xenopus*, gar, and coelacanth genomes (fig. 1). In the zebrafish *D. rerio*, the third round of teleost-specific genome duplication led to two *Col10a1* copies located on chromosomes 17 (*col10a1a*) and 20 (*col10a1b*), the first one located 14 kb upstream of one copy of *Frk* (*fyna* ENSDARG00000011370), the latter is located in the intron 6 of *nt5dc1* (ENSDARG00000006797) and 20 kb upstream of another copy of Frk (*fynrk* ENSDARG00000027807).

We first focused on intron 6 of *Nt5dc1* in the elephant shark *C. milii* to undergo the Genscan analysis that led to the identification of five consecutive *Col10a1* genes (fig. 1). We further analyzed the 85 kb long intergenic region between the first exon of *Frk* and the first exon of *Nt5dc1* but found no *Col10a1* copy (this region however also contains a sequencing gap). In the whale shark *R. typus*, strong similarity was detected in several predicted transcripts: LOC109931113; LOC109919892 located 30 kb upstream of a *Frk* gene (Gene ID: 109919891); LOC109914588 which is a composite predicted transcript including four consecutive *Col10a1* sequences, and is located in the intron 6 of *Nt5dc1* (LOC109914587, fig. 1). The cDNA sequences from the catshark transcriptome were used as BLAST queries against a partial catshark genomic assembly (Oulion et al. 2010) and could be located on different short scaffolds. One scaffold confirmed the synteny of one copy with the six first *Nt5dc1* exons, and another scaffold showed the tandem position of the *Col10a1.3* and *Col10a1.4* copies (fig. 1). As no fully assembled batoid genome is currently available, and to better support synteny of the tandem copies in elasmobranchs, we used cDNA sequences from the thornback ray and little skate as BLAST queries against the draft genome sequence of the thorny skate *Amblyraja radiata* (assembly version sAmbRad1_p1): exons of all six tandem gene copies of *Col10a1* could be identified along a 400 kb long portion of the scaffold 000143F in synteny with *Frk* and *Nt5dc1* genes (fig. 1). The data obtained in whale shark and thorny skate genomes highlights the position of the *Col10a1.1* copy, not in the intron 6 of *Nt5dc1* but in the intergenic space between the start of genes *Frk* and *Nt5dc1* (fig. 1).

### Phylogeny and Evolutionary History of *Col10a1* Tandem Duplications

**Fig. 2.**
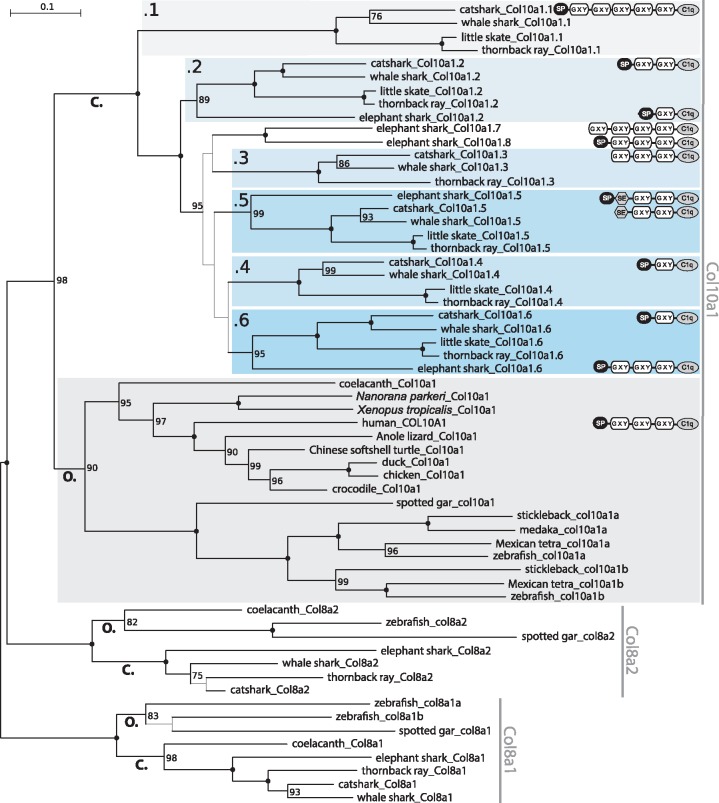
Maximum likelihood phylogenetic tree based on analysis of *Col10a1* nucleotide sequences with GTR+R4+F evolution model in IQ-TREE. Bipartition support was evaluated by 100 bootstrap replicates. Maximum support is shown by filled circles. Bootstrap supports below 75 are not displayed, and the branch thickness of their corresponding bipartition was decreased. *Col8a1* and *Col8a2* genes were used as outgroups and tree was rooted following Aldea et al. (2013). Predicted protein domains for catshark, elephant shark, and human Col10a1 sequences are displayed next to the gene names. Protein domains are labeled as SP—signal peptide, GXY—collagen triple alpha helix repeat, C1q—complement component 1q domain, and SE—serine-rich, acidic domain. Abbreviations: C., *Chondrichthyes* (cartilaginous fishes); O., *Osteichthyes* (bony fishes).

To better describe the evolutionary history of tandem duplications, we used these *Col10a1* sequences from osteichthyan and chondrichthyan species, together with their paralogues *Col8a1* and *Col8a2*. Our phylogenetic reconstructions were rooted with the *Col8a1* clade (fig. 2 and supplementary fig. 1, Supplementary Material online). The jawed vertebrate *Col10a1* group was recovered monophyletic with strong support. Within it, all osteichthyan *Col10a1* sequences grouped together and were sister-group to the monophyletic group of the chondrichthyan *Col10a1* sequences. The branching between several paralogous groups suggests at least five local duplication events that most probably occurred in the chondrichthyan stem lineage as four out of six clades include both elasmobranch and holocephalan sequences. We chose to name the tandem gene duplicates by adding a number, from .1 to .8, following the original gene name *Col10a1*, with respect to the time of divergence (following the phylogenetic reconstruction [fig. 2] and their respective position along the chromosome [fig. 1]). The first duplication led to the *Col10a1.1* copy located in the *Nt5dc1–Frk* intergenic sequence and later lost in holocephalans, whereas its sister copy remained in the intron 6 of *Nt5dc1* (fig. 1, supplementary fig. 2, Supplementary Material online). A second duplication event led to the *Col10a1.2* copy kept in all analyzed chondrichthyans. A third event led to several tandem duplications for which little to no phylogenetic signal seems to remain as shown by the low support for their respective relationship (fig. 2): *Col10a1.5* and *Col10a1.6* are found in all analyzed chondrichthyan species whereas *Col10a1.3* and *Col10a1.4* are only well identified in elasmobranchs. Two additional elephant shark copies cluster together, possibly suggesting additional tandem duplication in the holocephalan lineage, therefore, these genes are currently named *Col10a1.7* and *Col10a1.8* (fig. 2).

### Protein Domain Organization of Col10a1 Duplicates in Chondrichthyans

Protein domain predictions showed that the elephant shark and catshark Col10a1 duplicates share typical domain architecture and organization with osteichthyan Col10a1 proteins (fig. 2). Missing signal peptides in some sequences can be explained by incompleteness of genomic and cDNA data. In both holocephalans and elasmobranchs we detected an additional domain in Col10a1.5, located after the signal peptide and before the triple alpha helix repeat (fig. 2). This domain (named SE) is rich in serine (S), glutamic acid (E), and also to some degree in aspartic acid (D) (supplementary fig. 3, Supplementary Material online). Conserved domain analysis annotated this domain as DMP1 superfamily cl25845 and pfam07263 (Marchler-Bauer et al. 2017). This superfamily includes dentin matrix acidic phosphoprotein 1 (DMP1) which has a similar composition of amino acids. For example human DMP1 has the highest number of S (21%), E (15%), and D (13%). In comparison, SE domains in Col10a1.5 of catshark, whale shark, thornback ray, and elephant shark contain the highest number of S (21–31%) and E (10–12%) but lower number of D (5–8%) (supplementary fig. 3, Supplementary Material online). In addition we have discovered a short domain S/D S/D DSSEE in Col10a1.2 and another short domain SE E/D XXXEXX E/D E/D XX E/D in Col10a1.4. Both these domains are located in the similar position with the SE domain of Col10a1.5 after the signal peptide and before the triple alpha helix repeat (supplementary fig. 4, Supplementary Material online).

### Specialized Expression Patterns of Duplicated *Col10a1* Genes in Elasmobranchs

From the transcriptome data of the catshark lower jaw, the catshark vertebra and thornback ray lower jaw, we presented *Col10a1* gene expression values as Transcripts Per Million (TPM). In lower jaws all six tandem duplicates were expressed, with values above 10 detected for *Col10a1.1*, *Col10a1.2*, *Col10a1.4*, and *Col10a1.6* in catshark and ray, as well as for *Col10a1.3* in catshark (table 1). In contrast, in catshark vertebra only *Col10a1.4* had a value above 10, although *Col10a1.2* also showed detectable levels below 10.

**Table 1.** *Col10a1* Gene Expression Levels in Transcripts Per Million Reads Sequenced (TPM) Obtained from Transcriptome Analysis of Catshark Lower Jaw and Vertebra, and Thornback Ray Lower Jaw.

Gene Name	Lower Jaw (TPM)	Vertebra (TPM)
Catshark_*Col10a1.1*	12	<1
Thornback ray_*Col10a1.1*	10	—
Catshark_*Col10a1.2*	31	7
Thornback ray_*Col10a1.2*	16	—
Catshark_*Col10a1.3*	11	<1
Thornback ray_*Col10a1.3*	2	—
Catshark_*Col10a1.4*	110	194
Thornback ray_*Col10a1.4*	92	—
Catshark_*Col10a1.5*	5	<1
Thornback ray_*Col10a1.5*	8	—
Catshark_*Col10a1.6*	18	<1
Thornback ray_*Col10a1.6*	77	—

**Fig. 3.**
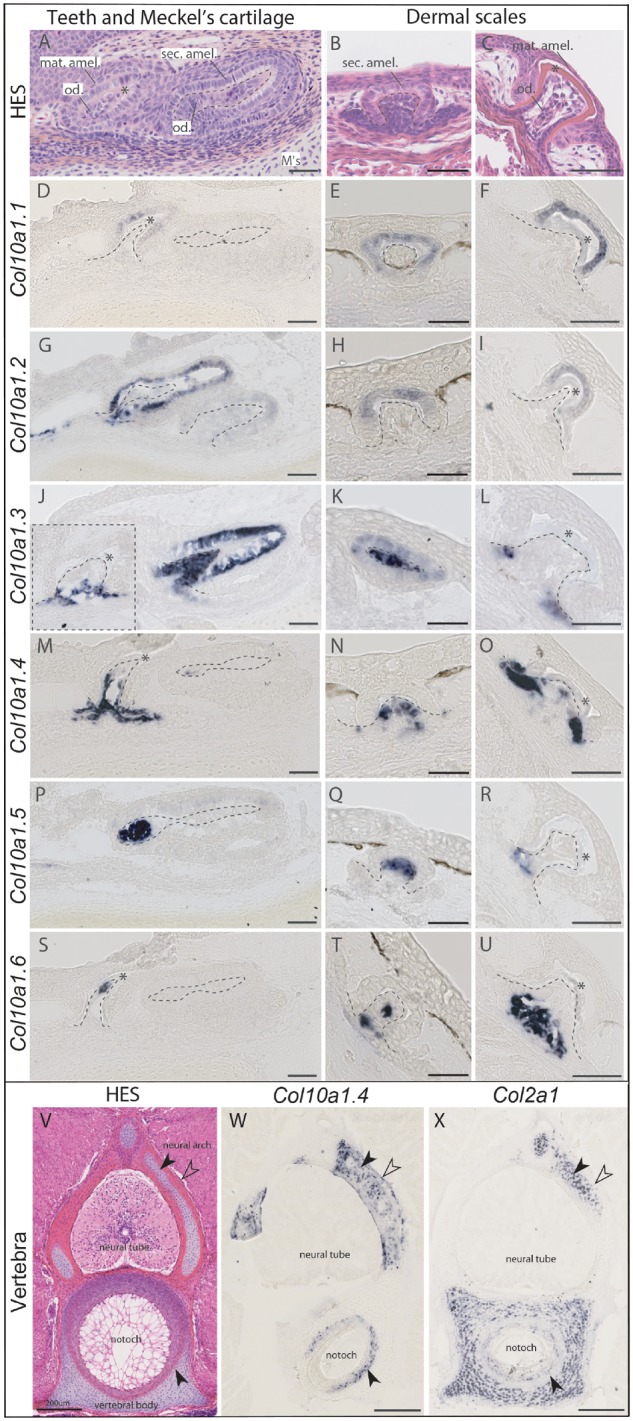
Histology and in situ hybridization on cryosections of catshark *Scyliorhinus canicula* skeletal tissues at stage 33. **(***A*, *B*, *C*, *V*) Hematoxylin–Eosin–Saffron (HES) histological staining. (*D*–*U*) Expression of *Col10a1* duplicates in developing teeth and scales of catshark detected with probes specific for each duplicate. Dotted lines indicate the mesenchymal compartment limits. Asterisk marks the mineralizing matrix, often seen as empty space due to decalcification. (*W*, *X*) Expression of *Col10a1.4* duplicate in comparison to *Col2a1* in mineralizing vertebral tissues of catshark. Black arrowhead in the neural arch points to the site of globular mineralization; open arrowhead points to the site of lamellar mineralization; black arrowhead in the vertebral body points to the site of fibrous mineralization. Scale bars: 50 µm (*A*–*U*), 200 µm (*V*–*X*). Abbreviations: mat. amel., maturation stage ameloblasts; M’s, Meckel’s cartilage; notoch, notochord; od., odontoblasts; sec. amel., secretory ameloblasts.

To further locate the detailed gene expression pattern of each duplicate, we performed in situ hybridization with RNA probes specific to each copy of catshark *Col10a1* (fig. 3). In jaw sections, we focused on successive stages of tooth development showing in the epithelial compartment: (i) the columnar secretory ameloblasts as identified by the presence of translucent secretory vesicles at their secretory pole (fig. 3*A* and *B*) and (ii) the maturation stage with cubic ameloblasts, devoid of secretory vesicles in their cytoplasm, facing a mineralizing extracellular matrix (fig. 3*A* and *C*). At these stages, the mesenchymal compartment is composed of differentiating and secretory odontoblasts (fig. 3*A*). Transverse sections through the abdomen allowed observation of dermal scale development with secretory ameloblasts facing undifferentiated mesenchymal cells (fig. 3*B*), and of maturation ameloblasts facing secretory odontoblasts at a later stage of scale development (fig. 3*C*).

All six duplicates were expressed in developing teeth with various expression patterns. *Col10a1.1* and *Col10a1.2* were transcribed in the epithelium of developing teeth, at two successive stages of ameloblasts differentiation: the secretory and maturation stages (fig. 3*D* and *G*) whereas *Col10a1.4*, *Col10a1.5*, and *Col10a1.6* were transcribed in the mesenchymal cells of developing teeth as they differentiate to secretory odontoblasts (fig. 3*M*, *P*, and *S*). The *Col10a1.3* duplicate showed expression in both the epithelium (ameloblasts) and mesenchyme (odontoblasts) at the secretory stage of developing tooth buds (fig. 3*J*), but its expression was later restricted to the odontoblasts of older tooth buds (fig. 3*J*, dashed square). These expression patterns in teeth were comparable to the ones in developing scales on abdominal transverse sections with restricted expression of *Col10a1.1* and *Col10a1.2* in the secretory and maturation ameloblasts (fig. 3*E*, *F*, *H*, and *I*), restricted expression of *Col10a1.4*, *Col10a1.5*, and *Col10a1.6* in the mesenchymal compartment before (fig. 3*N*, *Q*, and *T*) and after odontoblasts differentiation (fig. 3*O*, *R*, and *U*). Similar to teeth, *Col10a1.3* showed expression in the mesenchymal compartment of developing scales, with transient expression in secretory ameloblasts (fig. 3*K* and *L*). No expression of any duplicate could be detected in the Meckel’s cartilage at this stage of development, when cartilage mineralization is known to be initiated (Enault et al. 2015).

On the same abdominal transverse sections, only *Col10a1.4* was detected in vertebral tissues during their mineralization (fig. 3*W*) in accordance with TPM values from the transcriptome data (table 1). Expression of the Col10a1.2 duplicate could not be detected in vertebral tissues (data not shown) despite low TPM values for this gene in the vertebral column transcriptome. We consider that little amounts of skin tissue including scales in RNA-seq samples might be the reason for these TPM results. The sites of mineralization described in catshark vertebrae (Enault et al. 2015) include the ring of fibrocyte-like cells, involved in areolar mineralization surrounding the notochord, the fibrous tissues surrounding the cartilaginous neural arches and the cartilaginous core of the neural arches, not mineralized at that stage but known to later be invaded by globular mineralization (Debiais-Thibaud 2019) (fig. 3*V* and *X*). *Col10a1.4* expression was detected in each of these mineralizing sites of vertebrae (fig. 3*W*) but not in the unmineralized cartilaginous part of the vertebral body which is positive for type II collagen expression.

### Novel Sites of *Col10a1* Expression in Two Osteichthyan Species

**Fig. 4.**
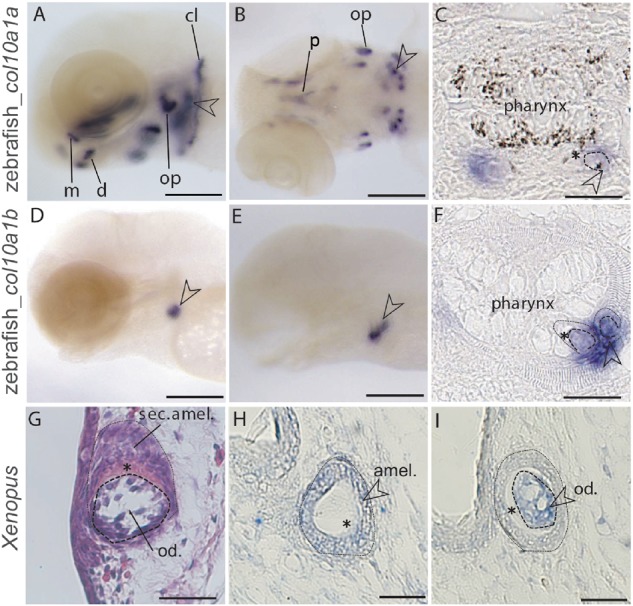
Expression of zebrafish *col10a1a* and *col10a1b* and *Xenopus Col10a1* during tooth development detected with in situ hybridization. (*A*, *B*) *col10a1a* expression in whole-mount zebrafish larvae at 4 and 6 days post fertilization (dpf). (*C*) *col10a1a* expression in post in situ paraffin sections of zebrafish larvae at 5 dpf. (*D*, *E*) *col10a1b* expression in whole-mount zebrafish larvae at 4 and 6 dpf. (*F*) *col10a1b* expression in post in situ paraffin sections of zebrafish larvae at 4 dpf. (*G*) HE staining of a longitudinal section through a developing tooth bud of a *Xenopus* larva at stage NF57. (*H*, *I*) *Col10a1* expression in *Xenopus* tooth bud on successive transversal sections. Open arrowheads point at developing pharyngeal teeth and asterisk marks the mineralizing matrix. Scale bars: 100 µm (*A*, *B*, *D*, *E*), 50 µm (*C*, *F*), 20 µm (*G*, *H*, *I*). Abbreviations: amel., ameloblasts; cl, cleithrum; d, dentary; m, maxilla; od., odontoblasts; op, opercle; p, parasphenoid; sec. amel., secretory ameloblasts.

We performed in situ hybridizations in the zebrafish *D. rerio* to test the expression of teleost-specific duplicates *col10a1a* and *col10a1b* during larvae development. Expression of *col10a1a* was detected in bones (e.g., dentary, maxilla, ethmoid plate, opercle, and cleithrum) of the zebrafish larvae (fig. 4*A* and *B*). Additional expression sites were detected in pharyngeal teeth at days 4, 5, and 6 of zebrafish development figure 4*A*–*C*. Expression of *col10a1b* was detected only in teeth at days 4 and 6 (fig. 4*D*–*F*). Post in situ paraffin sections reveal the expression of *col10a1a* and *col10a1b* in the mesenchyme of the older tooth (with deposited matrix) and the expression of *col10a1b* in the epithelium of a less developed tooth bud (without deposited matrix, fig. 4*C* and *F*).

In transversal teeth sections of *Xenopus Col10a1* expression was detected both in mesenchyme (odontoblasts) and epithelium (ameloblasts) (fig. 4*G*–*I*).

## Discussion

Our analysis of genome and transcriptome data from a variety of chondrichthyan species led to the identification of five *Col10a1* gene duplicates in holocephalans (elephant shark), and six *Col10a1* gene duplicates in elasmobranchs (lesser spotted catshark, whale shark, thornback ray, little skate, and thorny skate). This is a first reported characterization of the *Col10a1* gene orthologues outside of the osteichthyan group, which supports the origin of *Col10a1* before the divergence of osteichthyans and chondrichthyans (dated to 422–463 Ma, Benton et al. 2009). In addition, synteny and phylogenetic data (figs. 1 and 2) support an early timing of these duplications, before the divergence between holocephalans and elasmobranchs (dated to 352–447 Ma, Benton et al. 2009), with little subsequent rearrangement.

Our data suggest that *Col10a1.1* and *Col10a1.4* might have later been lost in the holocephalan lineage, although they may be missing simply because of incomplete genome sequencing. A putative secondary duplication of *Col10a1.3* might also be interpreted from our synteny data and phylogenetic reconstruction, with elephant shark *Col10a1.7* and *Col10a1.8* duplicates clustering in a sister-group to the elasmobranch *Col10a1.3* sequences. However this topology is poorly supported with nucleotide sequence, and not recovered in the analysis of the protein sequences (supplementary fig. 1, Supplementary Material online) so we chose to name them independently. The identified chondrichthyan duplications mostly occurred as tandem duplications at the locus conserved for the *Col10a1* gene in bony fishes, except for the first event leading to the *Col10a1.1* copy, which is found outside on the *Ntd5dc1* intron 6, however still located in synteny, close to the *Frk* gene (fig. 1 and supplementary fig. 2, Supplementary Material online). The initial conservation of such tandem duplicates might be explained by a positive selection for a higher copy number (Hahn 2009; Schrider and Hahn 2010; Margres et al. 2017) given the importance of high protein quantity in the synthesis of extracellular matrix to be provided during development. However, based on our expression data in shark, the long-term conservation of these copies seems to be due to subsequent subfunctionalization including the acquisition of specialized expression patterns and/or protein function.

The expression of all six catshark *Col10a1* gene duplicates in the developing teeth and scales first brings up their importance for the chondrichthyan odontode skeleton development, greater than their involvement in endoskeletal genesis. We discovered several levels of potential functional diversification represented by epithelial, epithelial–mesenchymal, and mesenchymal gene expression. Three duplicates *Col10a1.1, .2*, and *.3* showed transcription in ameloblasts of the catshark teeth and scales: these cells are secretory and/or maturation (mineralizing) ameloblasts (Kemp 1985). Previous evidence supports the ectodermal origin of the mineralized components of the shark tooth enameloid however there is an ongoing discussion whether organic component is secreted by ameloblasts or odontoblasts, or both (Kemp 1985). Taken together, our data suggest the involvement of catshark *Col10a1.1*, *Col10a1.2*, and *Col10a1.3* in the enameloid formation that had never been shown before for nonfibrillar collagens of type X.

On the other hand we describe four of the duplicated genes (*Col10a1.3* to *Col10a1.6*) with expression in mesenchymal cells of developing teeth and scales in the catshark. Interestingly, patterns in the mesenchymal compartment showed variation—pulp of the tooth and scale for *Col10a1.6* versus extensive expression in the root region for *Col10a1.3* and *Col10a1.4*. Nevertheless, these are difficult to interpret, as the dynamics of odontoblasts and adjacent cell type differentiation have not been studied in detail in chondrichthyan fishes. Still, the expression of *Col10a1.5* seems to be concentrated to the secretory odontoblasts indicating the involvement in the production of dentin matrix.

We questioned protein function for these various duplicates, by predicting protein domains, and identified collagenous domains with length variation between duplicates. In model organisms, type X collagen was linked to biomineralization processes through its ability to bind with annexins from the matrix vesicles that mediate apatite precipitation in the extracellular matrix (Kirsch and Wuthier 1994 and reviewed by Bottini et al. 2018). However the exact functions of the type X collagen protein domains in this process remain unknown and we can only speculate on these functions in chondrichthyans.

Unexpected identification of a novel serine-rich acidic domain in Col10a1.5 with similarity to DMP1 could indicate the combined role for this duplicate in the deposition of both acidic and collagenous matrix in the chondrichthyan dentin. DMP1 belongs to the acidic subfamily of secretory calcium-binding phosphoproteins (SCPP) together with bone matrix proteins (Kawasaki 2009). Strikingly, SCPPs could not be identified in chondrichthyan genomes (Venkatesh et al. 2014; Enault et al. 2018). The appearance of the SE domain might therefore come from a convergent event of protein sequence evolution that allows structural similarity between dentin tissues of osteichthyan and chondrichthyan groups. Overall, our analysis showed that these genes that are transcribed during the odontode skeleton development arose through rapid tandem duplication events and underwent subsequent subfunctionalization acquiring some specific characteristics that are currently unique for chondrichthyan species.

**Fig. 5.**
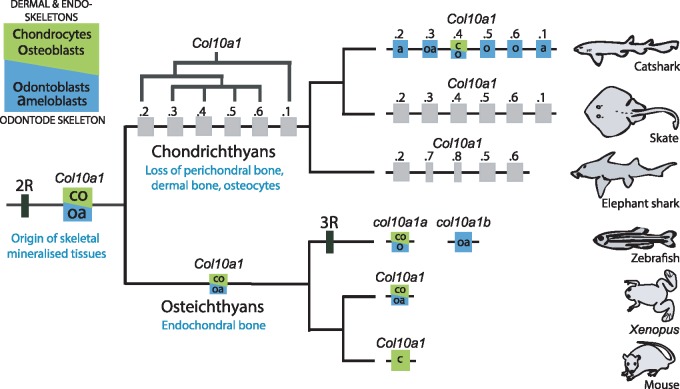
Proposed scenario for *Col10A1* locus evolution and subfunctionalization. *Col10a1* genes with expression in dermal skeleton and/or endoskeleton are marked as green boxes, with expression in odontode skeleton are marked as blue boxes and with unknown expression are marked by gray boxes. Tandem duplication of *Col10a1* genes in chondrichthyan ancestor is explained in detail in supplementary figure 2 (Supplementary Material online).

Analysis of *Col10a1* expression data only in catshark would have suggested that an ancestral type X collagen gene had been recruited to the odontode skeleton in the chondrichthyan lineage. However, a previous study reported *Col10a1* expression in zebrafish teeth (Simões et al. 2006) and our detailed analysis of *Col10a1* expression in zebrafish and *Xenopus* also revealed expression sites in the ameloblasts and odontoblasts of osteichthyan teeth. This supports that ameloblastic and odontoblastic expression of the *Col10a1* is a shared character of bony fishes (although apparently lost in mammals) and cartilaginous fishes, most probably inherited from the last common ancestor of all jawed vertebrates (fig. 5).

Because the osteichthyan type X collagen is a marker for osteoblasts and hypertrophic cartilage in osteichthyans, we questioned the importance of type X collagen for chondrichthyans where endochondral bone is missing. Only one duplicate (*Col10a1.4*) was strongly expressed during endoskeletal mineralization in the catshark. Expression was detected in standard chondrocytes going through globular mineralization, in “fibrocyte-like chondrocytes” of the fibrous vertebral body and in “osteocyte-like” cells that surround the neural arches. We did not detect expression of *Col10a1.4* or any other *Col10a1* duplicate in the Meckel’s cartilage, which is not mineralized yet, although the initiation of mineralization is set up at that developmental stage (Enault et al. 2015). We cannot completely exclude the possibility of convergent recruitment of type X collagen in several cell lineages and species lineages over vertebrate evolution, but this hypothesis involves many events: three independent recruitments in ameloblasts, odontoblasts, and mineralizing chondrocytes in early jawless vertebrates; osteoblasts in bony fishes; vertebral body chondrocytes in elasmobranchs and osteocyte-like cells in some shark species.

Instead, we propose an alternative scenario where the sharing of type X collagen gene expression by all these mineralizing cells is a hallmark of an ancestral biomineralization toolkit in early vertebrates that have been deployed in all mineralizing cells (fig. 5). Tandem duplication of type X collagen genes in chondrichthyan lineage was consequently followed by subfunctionalization of tandem duplicates. This process most probably required diversification of cis-regulatory mechanisms (Baudouin-Gonzalez et al. 2017). In this scenario, the regulatory elements linked to the ancestral *Col10a1* gene were activating expression in endoskeletal mineralizing cells, as well as ameloblasts and odontoblasts. These regulatory elements were then differentially inactivated between tandem duplicates so that only *Col10a1.4* kept the ancestral endoskeletal regulatory elements whereas all duplicates kept and also shared ameloblast and/or odontoblast specific regulatory elements.

As a final conclusion, we speculate here that the ancestral jawed vertebrate *Col10a1* gene originally belonged to a shared gene regulatory network linked to extracellular matrix mineralization. This toolkit was then initiated in a variety of cells found in the odontode skeleton, dermal skeleton, and endoskeleton of early vertebrates. Our analysis of *Col10a1* subfunctionalization after tandem duplications in chondrichthyans is a great example of how such toolkit can diversify during evolution. This scenario had never been explored before because of the lack of type X collagen expression by osteoblasts and odontoblasts in amniotes, in contrast to the situation of type I collagen which is involved in matrix secretion for dentin, bone, and even amphibian enameloid (Assaraf-Weill et al. 2014). However, type I collagen has no function in cartilaginous matrix and is never found associated to mineralized cartilage (Eames et al. 2007; Enault et al. 2015; Gómez-Picos and Eames 2015; Cervantes-Diaz et al. 2017). For these reasons, candidates for the description of the ancestral gene regulatory network linked to biomineralization should be chosen for their interaction with type X collagen, and be tested in ray-finned fishes and cartilaginous fishes rather than amniotes.

## Materials and Methods

### Embryo Collection and Ethics Statement

Embryos of the lesser spotted catshark *S. canicula* from a Mediterranean population were obtained at the Station Méditerranéenne de l’Environnement Littoral, Sète, France. Collection and handling of catshark embryos were carried out in full compliance of institutional, national, and international guidelines (European Communities Council Directive of 22 September 2010 [2010/63/UE]) and did not require further approval by an ethics committee. Embryos were raised in seawater tanks at 18 °C and euthanized by overdose of MS-222 (Sigma) at selected stages (according to Ballard et al. 1993). Zebrafish *D. rerio* embryos were collected at SciLifeLab Genome Engineering Zebrafish Facility in Uppsala, Sweden, which holds an ethical permit from the Swedish Board of Agriculture. The Ethics Committee of the University of Concepcion approved the experimental procedures used to euthanize and process *X. tropicalis* tadpoles, which were strictly performed following the recommendations of the Biosafety and Bioethics Manual of the National Commission of Scientific and Technological Research (CONICYT, Chilean Government).

### RNA Sequencing

A section of the abdomen including the vertebral column was dissected into RNAlater from one catshark embryo at stage 32 (3.5-month old). The sample was homogenized in liquid nitrogen and total RNA extracted with RNeasy fibrous tissue Mini kit (Qiagen). Total RNA quality and quantity were determined using Bioanalyzer 2100 and Qubit 3.0. The library was prepared from 360 ng total RNA using the TruSeq stranded mRNA library preparation kit, from which the Poly(A)+ mRNA fraction was isolated using poly-T oligo-attached magnetic beads, then chemically fragmented, and finally reverse transcribed to generate cDNAs that are ligated to adapters. These products were purified and amplified with PCR to generate the indexed final double stranded cDNA library. The quality of the library was evaluated in TapeStation 4200, High Sensitivity assay; the quantity of the library was analyzed by real-time PCR in LightCycler 480 (Roche). Three hundred and sixty nanograms of the cDNA library was sequenced by paired-end sequencing (100 × 2) in Illumina HiSeq 2500 sequencer.

Transcriptome sequencing data were deposited into NCBI SRA database under accession number SRR8753342.

### De Novo Transcriptome Assembly

Sequenced reads of catshark vertebral column as well as publicly available reads of catshark (SRR5179116 and SRR5179117) and thornback ray (SRR5114772) lower jaw transcriptomes (Irisarri et al. 2017) were assembled as follows. Reads were trimmed using Trimmomatic 0.36 (Bolger et al. 2014) under the following parameters: ILLUMINACLIP: TruSeq-PE.fa: 2: 30: 10 LEADING: 3 TRAILING: 3 SLIDINGWINDOW: 4: 15 MINLEN: 20. All catshark sequencing reads from different sequencing experiments were pooled together and high quality paired-end reads for each species were assembled with Trinity (Grabherr et al. 2011) using default parameters. Transcript expression levels were then estimated for each tissue using kallisto (Bray et al. 2016).

### Identification of Collagen Type X Genes in the Genomes and Transcriptomes of Chondrichthyans

Collagen 10a1 (*Col10a1*), Collagen 8a1 (*Col8a1*), and Collagen 8a2 (*Col8a2*) sequences for human, mouse, and zebrafish were collected from GenBank. Elephant shark whole genome assembly GCA_000165045.2 Callorhinchus_milii-6.1.3 (Venkatesh et al. 2014) was downloaded and screened for *Col10a1*, *Col8a1*, and *Col8a2* sequences using BLAST. Scaffolds with best matching alignments were downloaded and genes were predicted with Genscan (Burge and Karlin 1997) and Augustus (Stanke and Morgenstern 2005). Predicted Col10a1, Col8a1, and Col8a2 protein sequences from elephant shark were used as TBLASTN queries in little skate (*L. erinacea*) Transcriptomic Contigs-Build 2 of Skatebase (Wyffels et al. 2014) and against the locally assembled lesser spotted catshark and thornback ray jaw and vertebrae transcriptomes on the Montpellier Bioinformatics Biodiversity (MBB) platform (http://mbb.univ-montp2.fr/MBB//index.php). Nucleotide sequence data reported are available in the Third Party Annotation Section of the DDBJ/ENA/GenBank databases under the accession numbers TPA: BK010855-BK010872. Catshark *Col10a1* cDNA sequences were used to search for similar sequences in a whale shark genome (*R. typus*) using BLASTN (GCA_001642345.2 ASM164234v2 [Read et al. 2017]). Thornback ray and little skate cDNA sequences were used to map synteny on the Thorny skate *A. radiata* draft genome using BLAST (data accessed with permission from Vertebrate Genome Project https://vgp.github.io/genomeark/Amblyraja_radiata/).

### Phylogenetic Analysis

Predicted CDS and protein sequences for all identified collagen type X and type VIII genes from elephant shark, catshark, little skate, thornback ray, and whale shark together with sequences from osteichthyan species were used for phylogenetic tree reconstruction (supplementary table 1, Supplementary Material online). Sequences were aligned with Macse (Ranwez et al. 2011) in order to account for the codon structure, producing separate output files of aligned nucleotide and their corresponding amino-acid sequences. These alignments were then cleaned using hmmcleaner to mask unreliably aligned residues (https://metacpan.org/pod/HmmCleaner.pl). Final alignments used for subsequent phylogenetic reconstruction are available in supplementary figures 4 and 5 (Supplementary Material online).

Phylogenetic analyses were performed on both nucleotide and amino-acid alignments to infer the evolutionary history of these gene families and provide orthology assignments, by rooting the tree with the *Col8a1* clade (Aldea et al. 2013). This data set was used to reconstruct gene phylogenies in Maximum Likelihood using IQ-TREE 1.5.4 (Nguyen et al. 2015) under the GTR+R4+F evolution model for nucleotide data and under the LG4X+F evolution model for amino-acid data. Node support was estimated by performing a hundred bootstrap replicates for each analysis.

### Protein Domain Prediction

Conserved protein domains were predicted in the sequences of human, elephant shark, and catshark Col10a1 proteins using InterPro (Finn et al. 2017) and illustrated with Adobe Illustrator.

### Conserved Synteny Analysis

The synteny analysis was performed on the genomic region including the seven first exons of the *Nt5dc1* gene, and on the first exon of the *Frk* gene, which is the closest coding sequence to the exon 1 of *Nt5dc1* in tetrapods (fig. 1). Synteny data in osteichthyans was extracted from the Ensembl database for several genomes (human genome assembly: GRCh38.p10 [GCA_000001405.25]; gar genome assembly LepOcu1 [GCA_000242695.1]; coelacanth genome assembly LatCha1 [GCA_000225785.1]; zebrafish genome assembly GRCz10 [GCA_000002035.3]). The *X. tropicalis* 9.1 genomic data were visualized from XenBase (Karimi et al. 2018) where the *Col10a1* sequence mapped 3′ to the fifth exon of a gene named *Abhd10* which BLAST best hit is *Nt5dc1* in the mouse transcriptomic data. Homology between human exon 6 and *Lepisosteus* exon 9 was verified by CDS alignment. All sequence reference numbers are given in supplementary table 1 (Supplementary Material online). Genomic scaffolds containing identified *Col10a1* genes were obtained from genome databases of *C. milii* (Venkatesh et al. 2014) and *R. typus* (Read et al. 2017*).*The six individual *Col10a1* cDNA sequences from the catshark transcriptome were used to screen the latest genomic assembly of the catshark using BLASTN on the Galaxy platform on the MBB server (Oulion et al. 2010) which allowed to retrieve six contig sequences including one or more of the *Col10a1* cDNA sequences. Catshark *Frk* and *Nt5dc1* coding sequences were identified with TBLASTN searches of the homologous sequences from the elephant shark genome against our transcriptome (references in supplementary table 1, Supplementary Material online). Thornback ray and little skate *Col10a1* cDNA sequences were also used to identify synteny on the draft genome of the thorny skate *A. radiata* using BLASTN.

### In Situ Hybridization in Catshark

Identified catshark *Col10a1*, *Col8a1*, and *Col8a2* cDNA sequences were used to design following primers: Fw TGGAACACATTACTGCGGTAT and Rev CCTTTCAGACCTGATAGACC for *Sc-Col10a1.1 (*Sc-Col10a1F), Fw GAATGGTGCACTTCTGTTGC and Rev CACGATATCCTGCAACACCT for *Sc-Col10a1.2 (*Sc-Col10A), Fw TGTGGTTGGTAAACCAGGTC and Rev ACCGGGAAAACCTAATGGAC for *Sc-Col10a1.3 (*Sc-Col10C), Fw TCTGTGACTCTGGTCTATGG and Rev CCAGTTGCTCCTGCCTGTC for *Sc-Col10a1.4 (*Sc-Col10B), Fw CAACTATGATTCATCAAAAGG and Rev CAATTCAAGACGACACTTGCG for *Sc-Col10a1.5 (*Sc-Col10D), Fw GGATACAATGCTGCACGTCA and Rev ATAGCCATTTGTGCCTGCAG for *Sc-Col10a1.6 (*Sc-Col10E), Fw AAGGCCCATCTGGTTTACCT and Rev CTGTTCGGATGGAAGTTGCA for *Sc-Col8a1*, Fw GCTAGCCTTCATGTAGATGG and Rev CCTTAGGTAGAACATTGCTCA for *Sc-Col8a2.* Probes were designed on regions that include coding sequences. In order to test for potential cross-hybridization, we aligned the riboprobe sequences and calculated the identity matrix: the maximal values were 0.595 between *Col10a1.2* and *Col10a1.4*, 0.571 between *Col10a1.4* and *Col10a1.6*, and 0.562 between *Col10a1.2* and *Col10a1.6* (supplementary table 2 and fig. 6, Supplementary Material online). The observation that *Col10a1.4* is the only gene detected in vertebral tissue although being 59% identical with *Col10a1.2* and 57% identical with *Col10a1.6* in this matrix supports the fact that potential cross-hybridization is not a confounding factor in the interpretation of our results.

Sequences were amplified from cDNA extracted from the jaw of stage 33 embryos. PCR products were ligated into the pGEM-T easy vector using the TA cloning kit (Promega). Inserts were amplified using primers present in the vector backbone, and PCR products were used as a template to synthesize antisense DIG riboprobes in a 3 µl reaction containing 100–200 ng PCR product and using the DIG RNA labeling mix (Roche) and T7 or SP6 RNA polymerase (Promega) following manufacturer’s instructions. DIG-labelled riboprobes were purified on MicroSpin G50 column (GE Healthcare).

Whole embryos from stage 33 were fixed for 48 h in 4% PFA in 1× PBS at 4 °C and then transferred in 100% ethanol at −20 °C for storage. Catshark in situ hybridizations were performed on 14 µm thick cryosections (para-sagittal in lower jaws and transversal in the abdomen) and stored at −20 °C. In situ hybridization on sections was performed as described previously (Enault et al. 2015) with stringent conditions of hybridization at 70 °C. Images of the catshark in situ hybridization and histological staining were taken with Hamamatsu NanoZoomer 2.0-HT Slide Scanner (Montpellier RIO Imaging facility, INM Optique) with a 40× objective.

### In Situ Hybridization on Zebrafish and *Xenopus* Larvae

Primers were designed for zebrafish *col10a1a* Fw CCTGGAGCCAAAGGAGAGTT; Rev TATCGGCAGCAAAGACACCA; and zebrafish *col10a1b* Fw TTCATCTCCTGGGAAGCCTG; Rev TTCACCTCTGCTACCTGGTG gene sequences. Zebrafish cDNA was synthesized with SuperScript III First-Strand Synthesis Kit (ThermoFisher) and oligo dT primers using RNA isolated from zebrafish embryos at 5 days post fertilization (dpf) as a template. Fragments were PCR amplified and cloned from zebrafish cDNA into TOPO pCR II TOPO vector and antisense RNA probes were synthesized with either SP6 or T7 RNA polymerase and digoxigenin labeling mix (Roche). In situ hybridization on zebrafish whole-mount larvae was done as previously described (Filipek-Górniok et al. 2013). Whole-mount larvae after in situ were embedded in paraffin and sectioned at 6 µm. Sections were imaged with 40× objective on Leica DM5500B microscope and Hamamatsu NanoZoomer S60 Digital Slide Scanner.

In situ hybridization on paraffin sections of *X. tropicalis* stage NF60 tadpoles were performed using previously reported *Col10a1* probe and previously described protocol (Aldea et al. 2013).

## Supplementary Material

Supplementary data are available at *Molecular Biology and Evolution* online.

## Supplementary Material

msz145_Supplementary_Data

## References

[msz145-B1] Albertson RC, Yan Y-L, Titus TA, Pisano E, Vacchi M, Yelick PC, Detrich HW, Postlethwait JH. 2010 Molecular pedomorphism underlies craniofacial skeletal evolution in Antarctic notothenioid fishes. BMC Evol Biol. 10:4. 20053275 10.1186/1471-2148-10-4 PMC2824663

[msz145-B2] Aldea D, Hanna P, Munoz D, Espinoza J, Torrejon M, Sachs L, Buisine N, Oulion S, Escriva H, Marcellini S. 2013 Evolution of the vertebrate bone matrix: an expression analysis of the network forming collagen paralogues in amphibian osteoblasts. J Exp Zool B Mol Dev Evol. 320 6:375–384. 23677533 10.1002/jez.b.22511

[msz145-B3] Arratia G, Schultze HP, Casciotta J. 2001 Vertebral column and associated elements in dipnoans and comparison with other fishes: development and homology. J Morphol. 250 2:101–172. 11746457 10.1002/jmor.1062

[msz145-B4] Assaraf-Weill N, Gasse B, Silvent J, Bardet C, Sire J-Y, Davit-Béal T. 2014 Ameloblasts express type I collagen during amelogenesis. J Dent Res. 93 5:502–507. 24570147 10.1177/0022034514526236

[msz145-B5] Atake OJ, Cooper DML, Eames BF. 2019 Bone-like features in skate suggest a novel elasmobranch synapomorphy and deep homology of trabecular mineralization patterns. Acta Biomater. 84:424–436. 30500446 10.1016/j.actbio.2018.11.047

[msz145-B6] Ballard WW, Mellinger J, Lechenault H. 1993 A series of stages for development of *Scyliorhinus canicula* the lesser spotted dogfish (Chondrichthyes: Scyliorhinidae). J Exp Zool. 267 3:318–336.

[msz145-B7] Baudouin-Gonzalez L, Santos MA, Tempesta C, Sucena É, Roch F, Tanaka K. 2017 Diverse cis-regulatory mechanisms contribute to expression evolution of tandem gene duplicates. Mol Biol Evol. 34 12:3132–3147. 28961967 10.1093/molbev/msx237 PMC5850857

[msz145-B8] Benton MJ, Donoghue PCJ, Asher RJ. 2009 Calibrating and constraining molecular clocks In: Hedges SB, Kumar S, editors. The timetree of life. New York: Oxford University Press p. 35–86.

[msz145-B9] Bolger AM, Lohse M, Usadel B. 2014 Trimmomatic: a flexible trimmer for Illumina sequence data. Bioinformatics 30 15:2114–2120. 24695404 10.1093/bioinformatics/btu170 PMC4103590

[msz145-B10] Bottini M, Mebarek S, Anderson KL, Strzelecka-Kiliszek A, Bozycki L, Simão AMS, Bolean M, Ciancaglini P, Pikula JB, Pikula S. 2018 Matrix vesicles from chondrocytes and osteoblasts: their biogenesis, properties, functions and biomimetic models. Biochim Biophys Acta Gen Subj. 1862 3:532–546. 29108957 10.1016/j.bbagen.2017.11.005 PMC5801150

[msz145-B11] Bray NL, Pimentel H, Melsted P, Pachter L. 2016 Near-optimal probabilistic RNA-seq quantification. Nat Biotechnol. 34 5:525–527. 27043002 10.1038/nbt.3519

[msz145-B12] Burge C, Karlin S. 1997 Prediction of complete gene structures in human genomic DNA. J Mol Biol. 268 1:78–94. 9149143 10.1006/jmbi.1997.0951

[msz145-B13] Cervantes-Diaz F, Contreras P, Marcellini S. 2017 Evolutionary origin of endochondral ossification: the transdifferentiation hypothesis. Dev Genes Evol. 227 2:121–127. 27909803 10.1007/s00427-016-0567-y

[msz145-B14] Criswell KE, Coates MI, Gillis JA. 2017 Embryonic development of the axial column in the little skate, *Leucoraja erinacea*. J Morphol. 278 3:300–320. 28144984 10.1002/jmor.20637

[msz145-B15] Dean MN, Summers AP. 2006 Mineralized cartilage in the skeleton of chondrichthyan fishes. Zoology (Jena) 109 2:164–168. 16584875 10.1016/j.zool.2006.03.002

[msz145-B16] Debiais-Thibaud M. 2019 The evolution of endoskeletal mineralisation in chondrichthyan fish: development, cells and molecules In:Johanson Z, Underwood C, Richter M, editors. Evolution and development of fishes. Cambridge: Cambridge University Press p. 110–125.

[msz145-B17] Eames BF, Allen N, Young J, Kaplan A, Helms JA, Schneider RA. 2007 Skeletogenesis in the swell shark *Cephaloscyllium ventriosum*. J Anat. 210 5:542–554. 17451531 10.1111/j.1469-7580.2007.00723.x PMC2375745

[msz145-B18] Eames BF, Amores A, Yan Y-L, Postlethwait JH. 2012 Evolution of the osteoblast: skeletogenesis in gar and zebrafish. BMC Evol Biol. 12:27. 22390748 10.1186/1471-2148-12-27 PMC3314580

[msz145-B19] Enault S, Muñoz D, Simion P, Ventéo S, Sire J-Y, Marcellini S, Debiais-Thibaud M. 2018 Evolution of dental tissue mineralization: an analysis of the jawed vertebrate SPARC and SPARC-L families. BMC Evol Biol. 18 1:127. 30165817 10.1186/s12862-018-1241-y PMC6117938

[msz145-B20] Enault S, Muñoz DN, Silva WT, Borday-Birraux V, Bonade M, Oulion S, Ventéo S, Marcellini S, Debiais-Thibaud M. 2015 Molecular footprinting of skeletal tissues in the catshark *Scyliorhinus canicula* and the clawed frog *Xenopus tropicalis* identifies conserved and derived features of vertebrate calcification. Front Genet. 6:283. 26442101 10.3389/fgene.2015.00283 PMC4584932

[msz145-B21] Filipek-Górniok B, Holmborn K, Haitina T, Habicher J, Oliveira MB, Hellgren C, Eriksson I, Kjellén L, Kreuger J, Ledin J. 2013 Expression of chondroitin/dermatan sulfate glycosyltransferases during early zebrafish development. Dev Dyn. 242 8:964–975. 23703795 10.1002/dvdy.23981

[msz145-B22] Finn RD, Attwood TK, Babbitt PC, Bateman A, Bork P, Bridge AJ, Chang HY, Dosztanyi Z, El-Gebali S, Fraser M, et al 2017 InterPro in 2017-beyond protein family and domain annotations. Nucleic Acids Res. 45(D1):D190–D199. 27899635 10.1093/nar/gkw1107 PMC5210578

[msz145-B23] Gómez-Picos P, Eames BF. 2015 On the evolutionary relationship between chondrocytes and osteoblasts. Front Genet. 6:297. 26442113 10.3389/fgene.2015.00297 PMC4585068

[msz145-B24] Grabherr MG, Haas BJ, Yassour M, Levin JZ, Thompson DA, Amit I, Adiconis X, Fan L, Raychowdhury R, Zeng Q, et al 2011 Full-length transcriptome assembly from RNA-seq data without a reference genome. Nat Biotechnol. 29 7:644–652. 21572440 10.1038/nbt.1883 PMC3571712

[msz145-B25] Hahn MW. 2009 Distinguishing among evolutionary models for the maintenance of gene duplicates. J Hered. 100 5:605–617. 19596713 10.1093/jhered/esp047

[msz145-B26] Hall BK. 2015 Bones and cartilage. San Diego (CA): Elsevier.

[msz145-B27] Irisarri I, Baurain D, Brinkmann H, Delsuc F, Sire J-Y, Kupfer A, Petersen J, Jarek M, Meyer A, Vences M, et al 2017 Phylotranscriptomic consolidation of the jawed vertebrate timetree. Nat Ecol Evol. 1 9:1370–1378. 28890940 10.1038/s41559-017-0240-5 PMC5584656

[msz145-B28] Jacenko O, LuValle PA, Olsen BR. 1993 Spondylometaphyseal dysplasia in mice carrying a dominant negative mutation in a matrix protein specific for cartilage-to-bone transition. Nature 365 6441:56–61. 8361538 10.1038/365056a0

[msz145-B29] Janvier P. 1996 Early vertebrates. New York: Oxford University Press.

[msz145-B30] Karimi K, Fortriede JD, Lotay VS, Burns KA, Wang DZ, Fisher ME, Pells TJ, James-Zorn C, Wang Y, Ponferrada VG, et al 2018 Xenbase: a genomic, epigenomic and transcriptomic model organism database. Nucleic Acids Res. 46(D1):D861–D868. 29059324 10.1093/nar/gkx936 PMC5753396

[msz145-B31] Kawasaki K. 2009 The SCPP gene repertoire in bony vertebrates and graded differences in mineralized tissues. Dev Genes Evol. 219 3:147–157. 19255778 10.1007/s00427-009-0276-x PMC3713409

[msz145-B32] Kemp NE. 1985 Ameloblastic secretion and calcification of the enamel layer in shark teeth. J Morphol. 184 2:215–230. 3989869 10.1002/jmor.1051840211

[msz145-B33] Kirsch T, Wuthier RE. 1994 Stimulation of calcification of growth plate cartilage matrix vesicles by binding to type II and X collagens. J Biol Chem. 269 15:11462–11469. 8157677

[msz145-B34] Kwan KM, Pang MKM, Zhou S, Cowan SK, Kong RYC, Pfordte T, Olsen BR, Sillence DO, Tam PPL, Cheah KSE. 1997 Abnormal compartmentalization of cartilage matrix components in mice lacking collagen X: implications for function. J Cell Biol. 136 2:459–471. 9015315 10.1083/jcb.136.2.459 PMC2134813

[msz145-B35] Lallier F, Vidal B, Peignoux-Deville J, Lallier F, Vidal B. 1982 Evidence for the presence of osseous tissue in dogfish vertebrae. Cell Tissue Res. 222 3:605–614. 7060106 10.1007/BF00213858

[msz145-B36] Laue K, Jänicke M, Plaster N, Sonntag C, Hammerschmidt M. 2008 Restriction of retinoic acid activity by Cyp26b1 is required for proper timing and patterning of osteogenesis during zebrafish development. Development 135 22:3775–3787. 18927157 10.1242/dev.021238 PMC3608526

[msz145-B37] Marchler-Bauer A, Bo Y, Han L, He J, Lanczycki CJ, Lu S, Chitsaz F, Derbyshire MK, Geer RC, Gonzales NR, et al 2017 CDD/SPARCLE: functional classification of proteins via subfamily domain architectures. Nucleic Acids Res. 45(D1):D200–D203. 27899674 10.1093/nar/gkw1129 PMC5210587

[msz145-B38] Margres MJ, Bigelow AT, Lemmon EM, Lemmon AR, Rokyta DR. 2017 Selection to increase expression, not sequence diversity, precedes gene family origin and expansion in rattlesnake venom. Genetics 206 3:1569–1580. 28476866 10.1534/genetics.117.202655 PMC5500151

[msz145-B39] Mcintosh I, Abbott MH, Warman ML, Olsen BR, Francomano CA. 1994 Additional mutations of type X collagen confirm COL10A1 as the Schmid metaphyseal chondrodysplasia locus. Hum Mol Genet. 3 2:303–307. 8004099 10.1093/hmg/3.2.303

[msz145-B40] Nguyen LT, Schmidt HA, Von Haeseler A, Minh BQ. 2015 IQ-TREE: a fast and effective stochastic algorithm for estimating maximum-likelihood phylogenies. Mol Biol Evol. 32 1:268–274. 25371430 10.1093/molbev/msu300 PMC4271533

[msz145-B41] Ørvig T. 1951 Histologic studies of placoderms and fossil elasmobranchs. I: the endoskeleton, with remarks on the hard tissues of lower vertebrates in general. Ark Zool. 2:322.

[msz145-B42] Oulion S, Debiais-Thibaud M, d'Aubenton-Carafa Y, Thermes C, Da Silva C, Bernard-Samain S, Gavory F, Wincker P, Mazan S, Casane D. 2010 Evolution of Hox gene clusters in gnathostomes: insights from a survey of a shark (*Scyliorhinus canicula*) transcriptome. Mol Biol Evol. 27 12:2829–2838. 20616144 10.1093/molbev/msq172

[msz145-B43] Peignoux-Deville J, Bordat C, Vidal B, Bordatt C, Vidal B. 1989 Demonstration of bone cells in elasmobranchs: with osteoclasts resorbing. Tissue Cell 21 6:925–933. 18620290 10.1016/0040-8166(89)90043-8

[msz145-B44] Qu Q, Haitina T, Zhu M, Ahlberg PE. 2015 New genomic and fossil data illuminate the origin of enamel. Nature 526 7571:108–111. 26416752 10.1038/nature15259

[msz145-B45] Ranwez V, Harispe S, Delsuc F, Douzery E. 2011 MACSE: multiple alignment of coding SEquences accounting for frameshifts and stop codons. PLoS One 6 9:e22594. 21949676 10.1371/journal.pone.0022594 PMC3174933

[msz145-B46] Read TD, Petit RA, Joseph SJ, Alam MT, Weil MR, Ahmad M, Bhimani R, Vuong JS, Haase CP, Webb DH, et al 2017 Draft sequencing and assembly of the genome of the world’s largest fish, the whale shark: *Rhincodon typus* Smith 1828. BMC Genomics 18 1:532. 28709399 10.1186/s12864-017-3926-9 PMC5513125

[msz145-B47] Renn J, Winkler C. 2010 Characterization of collagen type 10a1 and osteocalcin in early and mature osteoblasts during skeleton formation in medaka. J Appl Ichthyol. 26 2:196–201.

[msz145-B48] Schmid TM, Linsenmayer TF. 1985 Developmental acquisition of type X collagen in the embryonic chick tibiotarsus. Dev Biol. 107 2:373–381. 3882482 10.1016/0012-1606(85)90319-7

[msz145-B49] Schrider DR, Hahn MW. 2010 Gene copy-number polymorphism in nature. Proc Biol Sci. 277 1698:3213–3221. 20591863 10.1098/rspb.2010.1180 PMC2981937

[msz145-B50] Seidel R, Blumer M, Pechriggl E-J, Lyons K, Hall BK, Fratzl P, Weaver JC, Dean MN. 2017 Calcified cartilage or bone? Collagens in the tessellated endoskeletons of cartilaginous fish (sharks and rays). J Struct Biol. 200 1:54–71. 28923317 10.1016/j.jsb.2017.09.005

[msz145-B51] Seidel R, Lyons K, Blumer M, Zaslansky P, Fratzl P, Weaver JC, Dean MN. 2016 Ultrastructural and developmental features of the tessellated endoskeleton of elasmobranchs (sharks and rays). J Anat. 229 5:681–702. 27557870 10.1111/joa.12508 PMC5055090

[msz145-B52] Simões B, Conceição N, Viegas CS, Pinto JP, Gavaia PJ, Hurst LD, Kelsh RN, Cancela ML. 2006 Identification of a promoter element within the zebrafish colXalpha1 gene responsive to runx2 isoforms Osf2/Cbfa1 and til-1 but not to pebp2alphaA2. Calcif Tissue Int. 79 4:230–244. 17033725 10.1007/s00223-006-0111-6

[msz145-B53] Stanke M, Morgenstern B. 2005 AUGUSTUS: a web server for gene prediction in eukaryotes that allows user-defined constraints. Nucleic Acids Res. 33(Web Server):W465–W467. 15980513 10.1093/nar/gki458 PMC1160219

[msz145-B54] Venkatesh B, Lee AP, Ravi V, Maurya AK, Lian MM, Swann JB, Ohta Y, Flajnik MF, Sutoh Y, Kasahara M, et al 2014 Elephant shark genome provides unique insights into gnathostome evolution. Nature 505 7482:174–179. 24402279 10.1038/nature12826 PMC3964593

[msz145-B55] Wurmbach H. 1932 Das wachstum des selachierwirbels und seiner gewebe. Zool Jahrb (Abt Anat Ont Tiere) 55:1–136.

[msz145-B56] Wyffels J, King BL, Vincent J, Chen C, Wu CH, Polson SW. 2014 SkateBase, an elasmobranch genome project and collection of molecular resources for chondrichthyan fishes. F1000Res. 3:191. 25309735 10.12688/f1000research.4996.1 PMC4184313

