# Supplementary material for: Skeletal Mineralization in Association with Type X Collagen Expression Is an Ancestral Feature for Jawed Vertebrates
Source: Mol Biol Evol. 2019 Jul 4;36(10):2265–76. doi: 10.1093/molbev/msz145 (PMC6759074; doi:10.1093/molbev/msz145)
Supplement: msz145_Supplementary_Data [file msz145_supplementary_data.zip › Supplementary Material Supplementary Table 1-2 Supplementary Figure 1-3.pdf]

Supplementary Material

Supplementary table 1. Accession numbers for *Col10a1, Col8a1* and *Col8a2* sequences.

Supplementary table 2. Nucleotide sequence identity matrix of catshark *Col10a1.1-Col10a1.6* riboprobes.

Supplementary figure 1. Phylogenetic reconstruction (ML) with amino-acid sequences. Species abbreviations: Ac (the anole lizard *Anolis carolinensis*), Am (the Mexican tetra *Astyanax mexicanus*), Ap (the duck *Anas platyrhynchos*), Cm (the elephant shark *Callorhinchus milii*), Cp (the crocodile *Crocodylus porosus*), Dr (the zebrafish *Danio rerio*), Ga (the stickleback *Gasterosteus aculeatus*), Gg (the chicken *Gallus gallus*), Hs (the human *Homo sapiens*), Lc (the coelacanth *Latimeria chalumnae*), Le (the little skate *Leucoraja erinacea*), Lo (the gar *Lepisosteus oculatus*), Np (the frog *Nanorana parkeri*), Ol (the medaka *Oryzias latipes*), Ps (the Chinese soft-shell turtle *Pelodiscus sinensis*), Sc (the catshark *Scyliorhinus canicula*), Rc (the ray *Raja clavata*), Rt (the whale shark *Rhincodon typus*), Xt (the clawed frog *Xenopus tropicalis*).

Supplementary figure 2. Proposed scenario of *Col10a1* gene (displayed as light blue boxes) duplications in cartilaginous fishes leading to genomic location and phylogenetic relationship in extant elasmobranchs.

Supplementary figure 3. Alignment of Col10a1.5 protein sequences from elephant shark (Cm), ray (Rc), catshark (Sc) and whale shark (Rt) constructed with AliView.

Supplementary figure 4. Amino acid sequence alignments used for subsequent phylogenetic analyses.

Supplementary figure 5. Nucleotide sequence alignments used for subsequent phylogenetic analyses.

Supplementary figure 6. Nucleotide sequence alignment of catshark *Col10a1.1-Col10a1.6* riboprobes.

Supplementary Table 1

SPECIES	Gene	ENSEMBL	NCBI	Scaffold ID	Position on scaffol	cds length (bp)
Anolis carolensis	Ac_Col10a1	ENSACAG00000012503, ENSACAT00000012493.2				
Astyanax mexicamus	Am_Col10a1a	ENSAMXG00000020866, ENSAMXT00000021477.2				
Astyanax mexicamus	Am_Col10a1b	ENSAMXG00000035187, ENSAMXT00000051234.1				
Anas platyrhynchus	Ap_Col10a1	ENSAPLG00000010244, ENSAPLT00000010661.1				
Crocodylus porosus	Cp_Col10a1		XM_019537941.1			
Danio rerio	Dr_col10a1b	ENSDARG00000101535, ENSDART00000162218				
Danio rerio	Dr_col10a1a	ENSDARG00000054753, ENSDART00000091021				
Danio rerio	Dr_col8a1a	ENSDARG00000077403, ENSDART00000102981				
Danio rerio	Dr_col8a1b	ENSDARG00000003533, ENSDART00000019846				
Danio rerio	Dr_col8a2	ENSDARG0000006089, 3ENSDART00000136811				
Gasterosteus aculeatus	Ga_Col10a1a	ENSGACG00000010232, ENSGACT00000013541.1				
Gasterosteus aculeatus	Ga_Col10a1b	ENSGACG00000006168, ENSGACT00000008176.1				
Gallus gallus	Gg_Col10a1	ENSGALG00000014965, ENSGALT00000024133.3				
Homo sapiens	Hs_Col10a1	ENSG00000123500:ENST00000327673				
Latimeria chalumnae	Lc-Col10a1		XM_006002932.2			
Latimeria chalumnae	Lc-Col8a1	ENSLACG00000015600, ENSLACT00000017842				
Latimeria chalumnae	Lc-Col8a2	ENSLACG00000017396, ENSLACT00000019923				
Lepisosteus oculatus	Lo_Col10a1	ENSLOCG00000016970, ENSLOCT00000021014				
Lepisosteus oculatus	Lo_Col8a1	ENSLOCG00000009451, ENSLOCT00000011553				
Lepisosteus oculatus	Lo_Col8a2	ENSLOCG00000001624, ENSLOCT00000001872				
Nanorana parkeri	Np_Col10a1		XM_018563595.1			
Oryzias latipes	Ol_Col10a1		XM_023953120.1			
Pelodiscus sinensis	Ps_Col10a1	ENSPSIG00000004859, ENSPSIT00000005236.1				
Xenopus tropicalis	Xt_Col10a1	ENSXETG00000033031, ENSXETT00000023573				
Callorhinchus milii, Elephant shark	Cm_Col10a1.2			scaffold 71	330669-336599	2355
Callorhinchus milii, Elephant shark	Cm_Col10a1.7			scaffold 71	363244-367988	2301
Callorhinchus milii, Elephant shark	Cm_Col10a1.8			scaffold 71	377435-386937	3099
Callorhinchus milii, Elephant shark	Cm_Col10a1.5			scaffold 71	392692-410074	2343
Callorhinchus milii, Elephant shark	Cm_Col10a1.6			scaffold 71	426399-437649	2562
Callorhinchus milii, Elephant shark	Cm_Col8a1	SINCAMT00000023174	XP_007893941	scaffold 42		
Callorhinchus milii, Elephant shark	Cm_Col8a2	SINCAMT00000004437	XP_007902444	scaffold 121		
Leucoraja erinacea, Little skate	Le_Col10a1.1			LSb2-ctg50203		
Leucoraja erinacea, Little skate	Le_Col10a1.2			LSb2-REVCOMPctg2668069		
Leucoraja erinacea, Little skate	Le_Col10a1.4			LSb2-REVCOMPctg41083		
Leucoraja erinacea, Little skate	Le_Col10a1.5			LSb2-REVCOMPctg68055		
Leucoraja erinacea, Little skate	Le_Col10a1.6			LSb2-REVCOMPctg688724		
Rhincodon typus, Whale shark	Rt_Col10a1.1		XM_020519917.1	NW_018040040.1		
Rhincodon typus, Whale shark	Rt_Col10a1.2		XM_020530447.1	NW_018064585.1		
Rhincodon typus, Whale shark	Rt_Col10a1.3			NW_018033689.1		
Rhincodon typus, Whale shark	Rt_Col10a1.4			NW_018033689.1		
Rhincodon typus, Whale shark	Rt_Col10a1.5			NW_018033689.1		
Rhincodon typus, Whale shark	Rt_Col10a1.6			NW_018033689.1		
Rhincodon typus, Whale shark	Rt_Col8a1		XM_020511015.1			
Rhincodon typus, Whale shark	Rt_Col8a2		XM_020520317.1			
Raja clavata, Thornback ray	Rc_Col10a1.1		BK010860			2756
Raja clavata, Thornback ray	Rc_Col10a1.2		BK010855			3303
Raja clavata, Thornback ray	Rc_Col10a1.3		BK010857			3340
Raja clavata, Thornback ray	Rc_Col10a1.4		BK010856			3033
Raja clavata, Thornback ray	Rc_Col10a1.5		BK010858			2878
Raja clavata, Thornback ray	Rc_Col10a1.6		BK010859			2383
Raja clavata, Thornback ray	Rc_Col8a1		BK010861			5270
Raja clavata, Thornback ray	Rc_Col8a2		BK010862			3221
Scyliorhinus canicula, Lesser spotted catshar	Sc_Col10a1.1		BK010865			2100
Scyliorhinus canicula, Lesser spotted catshar	Sc_Col10a1.2		BK010869			3289
Scyliorhinus canicula, Lesser spotted catshar	Sc_Col10a1.3		BK010870			1884
Scyliorhinus canicula, Lesser spotted catshar	Sc_Col10a1.4		BK010868			2956
Scyliorhinus canicula, Lesser spotted catshar	Sc_Col10a1.5		BK010867			1235
Scyliorhinus canicula, Lesser spotted catshar	Sc_Col10a1.6		BK010866			2396
Scyliorhinus canicula, Lesser spotted catshar	Sc_Col8a1		BK010863			5415
Scyliorhinus canicula, Lesser spotted catshar	Sc_Col8a2		BK010864			1817
Scyliorhinus canicula, Lesser spotted catshar	Sc_Frk		BK010871			4360
Scyliorhinus canicula, Lesser spotted catshar	Sc_Nt5dc1		BK010872			3160
Scyliorhinus canicula, Lesser spotted catshark			MK695500	scaffold624		
Scyliorhinus canicula, Lesser spotted catshark			MK695501	scaffold650		
Scyliorhinus canicula, Lesser spotted catshark			MK695502	scaffold4552		
Scyliorhinus canicula, Lesser spotted catshark			MK695503	scaffold9523		
Scyliorhinus canicula, Lesser spotted catshark			MK695504	scaffold23761		
Scyliorhinus canicula, Lesser spotted catshark			MK695505	scaffold25368		
Scyliorhinus canicula, Lesser spotted catshark			MK695506	scaffold34489		
Scyliorhinus canicula, Lesser spotted catshark			MK695507	scaffold217337		

Supplementary Table 2

Seq->	1.1	1.5	1.2	1.3	1.4	1.6
Col10a1.1-riboprobe	ID	0,266	0,441	0,294	0,447	0,438
Col10a1.5-riboprobe	0,266	ID	0,297	0,140	0,306	0,297
Col10a1.2-riboprobe	0,441	0,297	ID	0,253	0,595	0,562
Col10a1.3-riboprobe	0,294	0,140	0,253	ID	0,269	0,254
Col10a1.4-riboprobe	0,447	0,306	0,595	0,269	ID	0,571
Col10a1.6-riboprobe	0,438	0,297	0,562	0,254	0,571	ID

Supplementary Figure 1

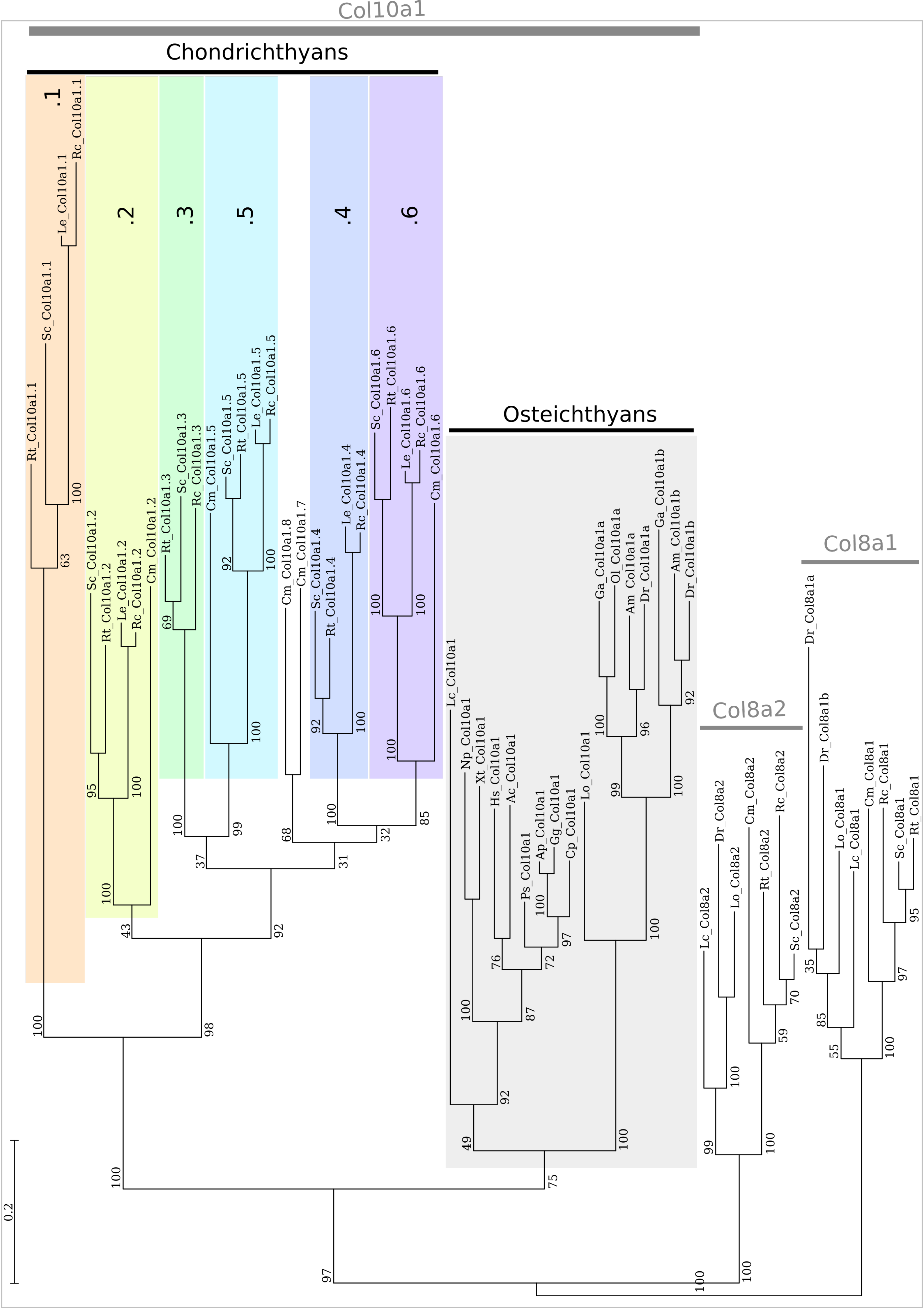

Col10a1
Chondrichthyans
.1
.2
.3
.5
.4
.6
Rt_Col10a1.1
Sc_Col10a1.1
Le_Col10a1.1
Rc_Col10a1.1
Sc_Col10a1.2
Rt_Col10a1.2
Le_Col10a1.2
Rc_Col10a1.2
Cm_Col10a1.2
Rt_Col10a1.3
Sc_Col10a1.3
Rc_Col10a1.3
Cm_Col10a1.5
Sc_Col10a1.5
Rt_Col10a1.5
Le_Col10a1.5
Rc_Col10a1.5
Cm_Col10a1.8
Cm_Col10a1.7
Sc_Col10a1.4
Rt_Col10a1.4
Le_Col10a1.4
Rc_Col10a1.4
Sc_Col10a1.6
Rt_Col10a1.6
Le_Col10a1.6
Rc_Col10a1.6
Cm_Col10a1.6
Osteichthyans
Lc_Col10a1
Np_Col10a1
Xt_Col10a1
Hs_Col10a1
Ac_Col10a1
Ps_Col10a1
Ap_Col10a1
Gg_Col10a1
Cp_Col10a1
Lo_Col10a1
Ga_Col10a1a
Ol_Col10a1a
Am_Col10a1a
Dr_Col10a1a
Ga_Col10a1b
Am_Col10a1b
Dr_Col10a1b
Col8a2
Lc_Col8a2
Dr_Col8a2
Lo_Col8a2
Cm_Col8a2
Rt_Col8a2
Rc_Col8a2
Sc_Col8a2
Col8a1
Dr_Col8a1a
Dr_Col8a1b
Lo_Col8a1
Lc_Col8a1
Cm_Col8a1
Rc_Col8a1
Sc_Col8a1
Rt_Col8a1
0.2

Supplementary Figure 2

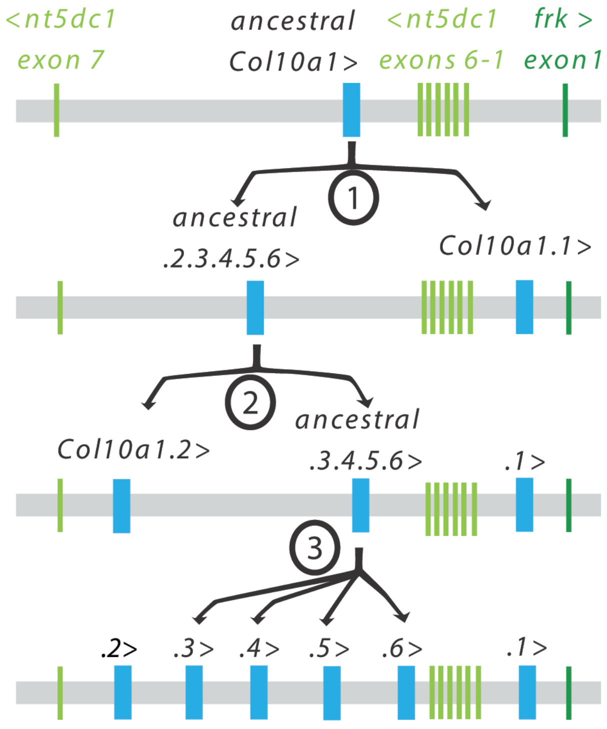

Ancestral chondrichthyan *Col10a1* duplicates

Supplementary Figure 3

```
                SE domain

                1
Cm-Col10A1.5    --------M ALKETSAFL LLLSITLVR ATG---YNR IFHNIKSYP GNEIHQHHS QSDETHSD- ---------
Rc-Col10A1.5    --------- --------- --------- --------- -----NDHS NSGSHQVKG QYSRRSS-- ---------
Sc-Col10a1.5    MRMCKLHTD SDPEPGSNL GPWCRKEVK GHD-----T PSNENNDDS KSASQQVKG QYSRRSS-- ---------
Rt-Col10a1.5    -DCCNMVSK G---IGALI LSLSLNLVY GTDFIRYVY PAYNIKSYG GVVSNFERL GYARVASGF TVNLPMNVK

                73
Cm-Col10A1.5    --------- --------- ---STSSSN EANNSEGYQ IKGHYSES- ----PEENS DSNSASHET S------DE
Rc-Col10A1.5    --------- --------- ----EENGN DHSNSGSHQ VKGQYSRR- --------- --------- --------S
Sc-Col10a1.5    --------- --------- ---EEKEQN GHSDSGSQQ VKGQYSRR- SSEEKEQNG HSDSGSQQV KGQYSRRSS
Rt-Col10a1.5    KLMVLCPEY KLTVTVISG CFYTAQIPD MVLNLGGLG NEVNFPHSL SNEDSEHNS NSKSASNEF KG-----NS

                145
Cm-Col10A1.5    SSNDSSDER SHERSHETK GYYSQITVG KGYNVDSSS SSSSSSEED GQSSESDSD SDSCSSESS SSSSSEENG
Rc-Col10A1.5    SEENGQNGN SNSGIYQVK GHYSRRSSS EE-NNQSTD ANSTSDESN DHDAEVNEE NNESDSSTT SSSSS----
Sc-Col10a1.5    SEEKEQNGH SDSGSHQIK GQYSRRSSS EE-NEQNGD ANSTSDESN GDDLEDIVG NNGSDYSSS SSSSS----
Rt-Col10a1.5    GPSDENNSD SNSASQQIK GQYSRRSSS EE-NQQNSD ANSTSDESN GHDLEDIVE NNNSDFSSS SSSSSSSSS

                217
Cm-Col10A1.5    GCHSSSS-- --------- --------- --------- -------SE EVSGEPGPP GP---PGPP GTQGYPGKS
Rc-Col10A1.5    --------- --------- --------- --------- --------E ERNGEPGPP GP---PGPY GPPGYPGKP
Sc-Col10a1.5    --------- --------- --------- --------- -------SE EHYGEPGPP GPPGIPGPH GPPGYPGKP
Rt-Col10a1.5    GLMFKTIYS KQAGAVGCE EKFFQEQTQ KESDLHNNL NGIYLFLTE EQYGEPGPP GPPGLPGPI GPPGYPGKP

                289
Cm-Col10A1.5    GYGMPGHPG KPGPRGPQG LSAMGKPGI PGASGKPGV PGNPGQRGD KGSQGSPGP RGPPGIKGT PGSNGLSIV
Rc-Col10A1.5    GFGLPGHPG KPGPPGPQG LSAVGKPGH PGETGRPGV PGSPGRKGD KGSPGKQAS RGPPGPSGP PGPAGLSVI
Sc-Col10a1.5    GFGLPGHSG KPGPPGPQG LSAIGKPGR PGEPGKSGV PGAPGRKGD KGTQGMQGP RGHQGPSGP PGPAGLSVI
Rt-Col10a1.5    GFGLPGHPG KPGPPGPQG LSAIGKPGG PGEPGKPGV PGSPGRKGD KGSQGMQGP RGPPGPSGP PGPAGLSVI

                361
Cm-Col10A1.5    GKPGAKGSS GSPGTIGFP GRKGDPGYP GLPGSKGAK GYGVPGSPG NKGPPGLQG PKGNPGPTG VGYPGPPGS
Rc-Col10A1.5    GKPGEKGEP GQPGTLGFP GRKGNPGYS GQPGPKGDK GVGSPGRPG DKGPPGPHG PPGPPGPEG KGHPGSPGS
Sc-Col10a1.5    GKPGAKGSP GPPGTLGFP GRKGDPGYA GLPGSKGDK GIGSPGRTG DKGVPGPRG PQGSPGADG KGHKGEHGQ
Rt-Col10a1.5    GKPGPKGSP GPPGTLGFP GRKGDPGYP GLPGPKGDK GVGSPGRPG DKGAPGPRG PQGPPGPEG KGLKGPPGH

                433
Cm-Col10A1.5    PGEPGKNGE KGKPGSSGQ AGIPGHKGL QGPPGIPGT GKPGSNGAK GSPGSPGSR GSSGPQGVP GAPGVPGIG
Rc-Col10A1.5    KGEPGHKGE KGSSGLPGE PGKQGQSGP AGPPGNQGI GKPGGRGPQ ---GIHGMK GHPGLQGLP GAPGLPGLG
Sc-Col10a1.5    KGEPGHKGE KGSPGSPGD PGKQGQTGK QGSPGKEGI GKPGIQGPR ---GVPGMK GHTGLQGLP GSPGLPGIG
Rt-Col10a1.5    KGEPGHKGE KGSPGSPGH PGKPGQDGP PGPPGKDGI GRPGIQGPR ---GVPGMK GHPGLQGLP GSPGLPGVG

                505
Cm-Col10A1.5    RPGERGSKG EPGSAGKIG KPGENGEKG SPGKPGAPG SPGSSGPQG PRGPKGQPG ANGATGEKG HPGLKGPSG
Rc-Col10A1.5    KPGLPGLKG HPGSPGETG KPGEKGKSG SPGAPGPRG LVGSPGHTG KNGPKGPPG TVGVPGEKG HPGIMGPKG
Sc-Col10a1.5    KPGLPGLKG HPGSPGLTG KPGDKGQKG SSGEPGPRG PAASPGPPG QKGPRGPPG NIGAPGVKG HPGLLGPHG
Rt-Col10a1.5    KPGLPGLKG HPGSPGLTG KPGDK---G SSGEPGPRG PVGPLGSPG PKGPKGPPG NVGAPGEKG HPGLRGPEG

                577
Cm-Col10A1.5    NPGSKGEPG PRGATGKPG STGDKGEPG SKGETGPVG KKGNAGPAG PPGKPGSPG AT---GPMG YPGEMGKSG
Rc-Col10A1.5    QMGLKGALG STGQQGPLG TPGKPGSDG HKGLPGPPG KNGRNGLIG QHGKPGSPG NAGPQGPQG HPGEPGKKG
Sc-Col10a1.5    SSGPKGEPG SKGEQGKSG SPGKQGEKG EKGESGSTG KKGDKGPNG PRGKAGDPG PPGPQGPQG HSGEPGKKG
Rt-Col10a1.5    PSGPKGEPG SKGEQGKPG PPGKSGAEG KKGAPGPSG EKGDKGPVG PRGKAGSPG PQGPQGPQG PPGKPGEKG

                649
Cm-Col10A1.5    APGSPGAKG SPGSHGAPG IPGEKGDPG IP---GPQG PPGAFNGKI VKGPPGAPG PGGTRGSPG HPGQPGPPG
Rc-Col10A1.5    HPGTPGSKG TSGLPGSPG HPGVKGDPG VPGTAGPSG PPGKFNGKV IMGPKGPPG PKGPGGPPG PPGQPGPAG
Sc-Col10a1.5    PPGQSGSVG STGSPGSPG HPGVKGDPG VP---GPPG SPGKCDAKT VKGPNGAPG PKGARGPSG IPGTPGPAG
Rt-Col10a1.5    HPGLPGSVG LTGSPGSPG HPGVKGDPG VP---GPPG PQSKFNAKI IKGPSGPPG PKGAIGPPG IPGAPGPAG

                721
Cm-Col10A1.5    PPGEMIMQP SKYSHHSSS -ESGESSDE NSTPAFTAI LSHPYPPTG KPIVFDKVL TNENENYDS STGIFTCEI
Rc-Col10A1.5    QPGEIIVHT GKYKGYNGY ITSGDVLSP V--PAFTAI LSQPYRSTG KPIVFDKIL TNANSNYDS SSGIFTCEI
Sc-Col10a1.5    PPGEIIMPK GKYAGHIGY IDSGDVVSP V--PAFTAI LSQPYPTKG KPIVFDKIL TNSNNNYDS SKGIFTCEI
Rt-Col10a1.5    PPGEIIIPK GKYAGYSGY IDSGGMVSP V--PAFTAI LSQPYPHKG QPIVFDKIL TNSNNNYDS SKGIFTCEI

                793
Cm-Col10A1.5    SGIYQFAYH VQVKSTSVL VGLYKNNEL VMITYDDYT KGYVDQASG SAILHLEED DKVYLQLPS EEFNGLYSS
Rc-Col10A1.5    TGIYQFFYH VHVKEANVW IGLYKNSEP VMYTYDEYS PQYVDHASG TAILHLEEG DKVYVKLPA EKSNGLYSS
Sc-Col10a1.5    SGIYQFFYH VHVKEANVW VALYKNDEA VMYTYDEYT PQYIDHASG TAILHLDEN DKVYVQLPA EKSNGVYSS
Rt-Col10a1.5    SGIYQFFYH VHVKEANAW VALYKNDEP VMYTYDEYT PQYVDHASG TAILHLDEN DKVYVQLPA EKSNGVYSS

                865
Cm-Col10A1.5    DEIYSSFSG FLIKSS--- --------- --------- --------- --------- --------- -----
Rc-Col10A1.5    EYMHSSFTG LLITPS--- --------- --------- --------- --------- --------- -----
Sc-Col10a1.5    EYMYSSFTG LLITPS--- --------- --------- --------- --------- --------- -----
Rt-Col10a1.5    EYIYSSFSG LLITPSIRS TTMVSKQIT VFLLLLTLN VIHGMGYNA AHQRLKGHG GSDSDGDDS DSSEG
```
